# Biological characterization of *Pasteurella multocida* present in the Saiga population

**DOI:** 10.1186/s12866-019-1407-9

**Published:** 2019-02-11

**Authors:** Mukhit Orynbayev, Kulyaisan Sultankulova, Abylay Sansyzbay, Rashida Rystayeva, Kamshat Shorayeva, Aidar Namet, Sasan Fereidouni, Gulnaz Ilgekbayeva, Kainar Barakbayev, Syrym Kopeyev, Richard Kock

**Affiliations:** 1 Research Institute for Biological Safety Problems, Ministry of Education and Science of Republic of Kazakhstan – Science Committee, 080409 Gvardeiskiy, Kordaiskiy rayon, Zhambylskaya oblast Republic of Kazakhstan; 2 Kazakh Scientific Research Veterinary Institute, 050016 Almaty, Raymbek Avenue 223, Republic of Kazakhstan; 3 Research Institute of Wildlife Ecology, University of Veterinary Medicine Vienna, Vienna, Austria; 4 Kazakh National Agrarian University, 050010 Almaty, Abai Avenue 8, Republic of Kazakhstan; 5 Royal Veterinary College, Hawkshead Lane, Hatfield, Herts AL9 7TA UK

**Keywords:** *Pasteurella multocida*, MLST, Saiga antelope, 16S rRNA gene, Virulence gene, Mass mortality events

## Abstract

**Background:**

This study provides biochemical and molecular genetic characteristics of *P. multocida* isolated from dead saigas in 1988, 2010–2015 on the territory of the Republic of Kazakhstan.

**Results:**

Bacteriological samples taken from carcasses of saiga antelope during mortality events recorded in West Kazakhstan in both 2010 and 2011 and in Kostanay in 2012 and 2015 confirmed the presence of *P. multocida,* according to morphological and biochemical characterisation. Only in the event of 2015 was the agent proven to be the causative agent of the disease observed, haemorrhagic septicaemia. In the other mortality events it is not certain if the organism was a primary aetiology or an incidental finding as confirmatory pathological investigation was not undertaken. The implemented phylogenetic analysis of ribosomal RNA 16S gene allowed us to identify *Pasteurella* strains isolated in 2010–2015 as *P. multocida subspecies multocida*. Capsular typing by PCR showed that the studied strains isolated from dead saiga in 2010, 2011, 2012 and 2015 belonged to serotype B. MLST analysis showed that these strains of *P. multocida* are of the capsule type B and form one clonal grouping with isolates ST64, ST44, ST45, ST46, ST44, ST47 which isolated from cases of hemorrhagic septicemia of animals in Hungary, Burma, Sri Lanka, Pakistan and Spain. Sixteen virulence genes of the five strains of *P. multocida*, isolated from saigas were studied using multiplex PCR. *ptfA, ompA, ompH, oma87, plpB, fimA, hsf-2, pfhA, exbB, tonB, hgbA, fur, nanB, nanH* and pmHAS genes were detected in all strains. The *toxA* gene was not identified in the studied strains. The phylogenies of these isolates is compared across saiga populations and years and the 2015 isolate was compared to that of an isolate from a disease outbreak in 1988 and the findings suggest that these isolated bacteria are stable commensals, opportunistically pathogenic, being phylogenetically uniform with very little genetic variation notable over the last 4 decades.

**Conclusion:**

Isolation, phenotypic and genetic characterization of the *P. multocida* isolates inform understanding of the epidemiology of infection in saigas and predict virulent potential of these opportunistic bacteria.

## Background

Bacteria of the Pasteurellaceae family can cause endemic and epidemic infectious diseases of domestic, wild animals and birds [1]. The most important members of the family Pasteurellaceae that pose a serious threat to animals are *Mannheimia haemolytica (M. haemolytica)*, *Pasteurella multocida* (*P. multocida*) and *Pasteurella trehalosi* (*Bibersteinia*) [2]. *P. multocida* is the most commonly reported pathogen of this family predominately causing respiratory disease in cattle and hoofed animals Haemorrhagic septicaemia (HS) is a specific, severe acute highly fatal prevalent disease in tropical geographies affecting mainly cattle and buffalo but there are reports in a number of other species affected and most usually with *P. multocida* serotype B2 [2]. A review [3] showed the widespread nature of HS, potential for latency in a herd, with bacteria present in nasopharyngeal secretions, intermittently and persisting in tonsillar crypts. Disease progression in farmed animals is rapid, with infection to death only a matter of hours to a few days and with both aerosol and oral infection routes demonstrated.

Pasteurellosis has been reported as a cause of disease of wild animals in Africa, America, Asia, Australia and Europe [4, 5]. Reports from free-ranging wildlife epidemics are rare and are from North America and Eurasia. The majority of cases reported in wild animals were under some form of management, including food supplementation on range, whilst in captivity or ex situ populations [6] and after translocation of captive Axis deer (*Axis axis*) and captive fallow deer *(Dama dama*) [7, 8]. Mass deaths of wild boars were also reported from Spain [9] where they are managed for purposes of hunting with supplementary feeding. Pathology of free-ranging mortality associated with pneumonic pasteurellosis has been most extensively studied in bighorn sheep where lesions are characterized by necrosis, haemorrhage and fibrinopurulent changes [10]. Cases of mass mortality of the saiga antelope were ascribed to pasteurellosis in Kazakhstan in 1981, 1984 and 1988 but extensive pathological diagnosis was not completed and it was not confirmed if these were due to HS, pneumonic pasteurellosis or involved other disease agents [11]. HS was also reported causing mass mortality of gerbils in the same ecosystem in Kazakhstan in 1959 and 1967 [12, 13]. In the former Turgay region approximately 70 thousand saiga died in May 1981 and approximately 270 thousand in May 1988 and in February and March 1984 between Volga and Ural rivers more than 110 thousand saigas died [14, 15]. Mass death of saiga was reported in spring in 2010 in West Kazakhstan (approximately 12,000 died) and this was followed in 2011 by a smaller die-off (approximately 500 died) but on the exact same location as in 2010 [16] and in 2012 in Kostanay region, Betpak-dala (approximately 1000 died) [17]. *P. multocida* was isolated from samples taken from a number of saigas, which died during all the above described outbreaks. In 2015, during calving in May another massive outbreak similar to that reported in 1988 in the same general location in Betpak-dala, occurred and was more fully investigated by Kazakh National and International veterinary scientists. Extensive monitoring and diagnostics confirmed, for the first time, a die-off due to HS caused by *P. multocida* serotype B. Significant co-infections were not found and a wide range of known virulent agents excluded whilst the epidemiology and environmental investigations suggested opportunistic infection from latent bacteria triggered by unusually humid and warm weather conditions prior to the mass mortality. These findings were consistent with earlier observations and retrospective modelling for the years 1981 and 1988 in addition to 2015 [18].

This report provides the first evidence as to which subspecies and serotypes *P. multocida*, isolated from the saigas in Kazakhstan belonged. Moreover, *P. multocida* virulence genes from these isolates had not been studied to date. Combination of virulence genes of strains and isolates of *P. multocida* bacteria may be different, but in all cases can provide a description of their pathogenicity.

This study provides biochemical and molecular genetic characteristics of *P. multocida* isolated from dead saiga samples taken in 1988, 2010–2015 in the Republic of Kazakhstan.

## Results

In May 2010 and 2015 mass mortality of saigas occurred during and after calving. In 2011 and 2012 smaller die-of events were observed. In all cases except 2015 the study of clinical signs of the disease and pathoanatomical changes was not possible due to delay in reaching the outbreak areas and extensive post mortem change. However, in all cases samples from dead saigas were obtained and laboratory tests were carried out in the Research Institute for Biological Safety Problems in Gvardeskiy (RIBSP) and in all cases *P. multocida* was detected. Isolates included Pasteurella/Saigas/ 2010/ZKO/KZ, 2011/ZKO/KZ, 2012/Kostanay/KZ, 2015/Kostanay/KZ, and 2015/Akmola/KZ, which are described in this study. No other known pathogens of ruminants were found of significance, by a variety of tests including Next Generation Sequencing of PCR products [18].

### Phenotypic characterization

#### Morphology

All five isolates of Pasteurella are short (0.5–1.5, 0.25–0.5 mm) coccovoids with rounded ends, stained negative by Gram. Bipolar coloration of cells can be seen when smears of the studied culture are stained by Romanovsky-Giemsa method [19].

#### Biochemical tests

Biochemical characterization of the isolates showed that they belonged to Pasteurella genus. All strains isolated from saigas were indole, catalase and oxidase positive, did not have hemolytic properties, lysed in bile, did not form hydrogen sulfide, reduced nitrates to nitrites, and were negative in Voges-Proskauer reaction. The isolates also showed positive reaction in urea hydrolysis, did not curdle sterile skim milk and showed a negative result in the determination of proteolytic bacteria properties such as liquefaction of gelatin and peptonization of milk protein - casein. The findings indicated that all five isolates were sorbitol-positive and dulcitol-negative, which is typical of the subspecies *P. multocida Ssp. multocida* [20].

### Molecular characterization

Molecular characterization showed that all the five studied strains belonged to *P. multocida* species.

### Сapsular PCR typing

**Fig. 1:**
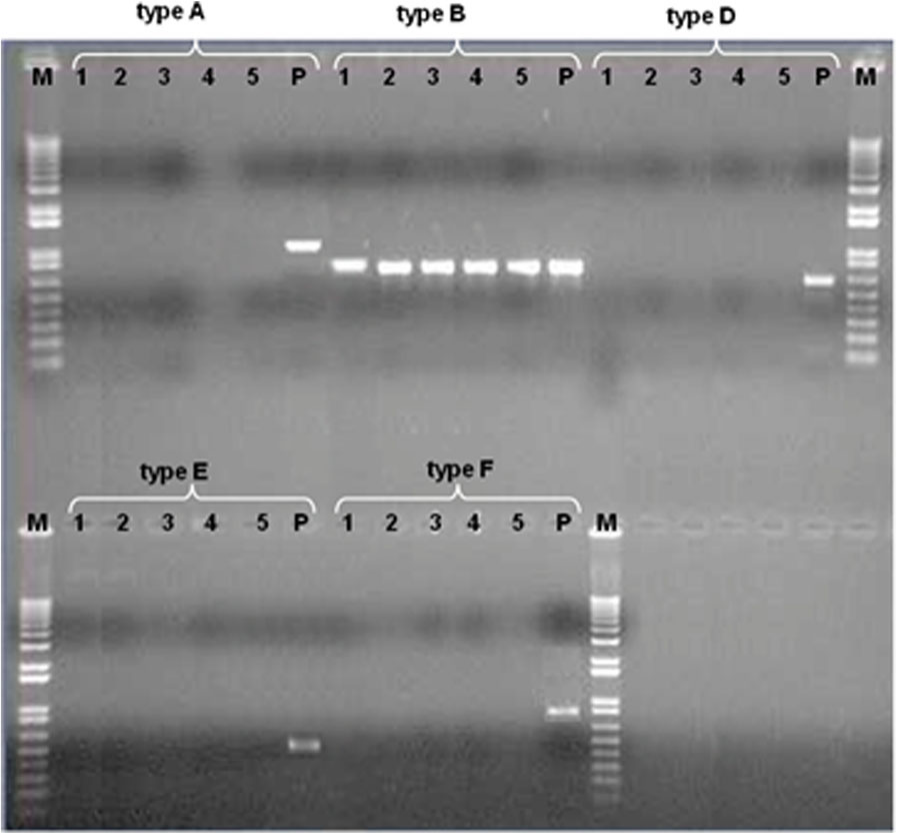
Capsular typing of the strains. Explanation*:* Figure showing positive controls working on all channels and consistent positive results for type B for the isolates: Pasteurella/Saigas/2010/ZKO/KZ (1), Pasteurella/Saigas/2011/ZKO/KZ (2), Pasteurella/Saigas/2012/Kostanay/KZ (3), Pasteurella/Saigas/2015/Kostanay/KZ (4) and Pasteurella/Saigas/2015/Akmola/KZ (5), М - 1 kb Marker, Invitrogen, P – type positive control

Capsular typing of the 2010–2015 isolates correspond to А, В, D, E, F capsular groups. The implemented studies resulted in amplification of PCR product of 760 bp characteristic for B serogroup shown in Fig. 1.

### *Pasteurella multocida* ribosomal RNA 16S analysis

The 16S rRNA gene carries both conservative and variable regions of the nucleotide sequence, which makes it possible to use it both for genotypes and for the species typing of microorganisms. The primers P16 Sf and P16 Sr were used and 1480 bp PCR products for all 5 isolates were obtained. The sequences were compared with the related sequences from the NCBI database, and phylogenetic dendrograms were constructed using the Mega 6.0 program.

Two isolates from 2015 outbreaks in Kostanay and Akmola were identical to the Saigachiy strain isolated in 1988, and all clustered together with *P. multocida* subsp.multocida_PM30 (AY299312), isolated from Bovine in 2004 in the same clade.

Comparison of complete sequences of the 16S rRNA gene isolates of *P. multocida* in 2010 with 1988, 2011, 2012 and 2015 showed that they differ by only one nucleotide.

### Multi locus sequence typing (MLST) analysis

Comparative analysis of nucleotide sequences using the online-program BLAST showed that all Kazakhstan strains are identical (100% homology) in all the studied genes.

MLST analysis showed that strains of Kazakhstan refers to the one group with clonal isolates of scheme ST64, 47, 46, 45, 44, 61 (Table 1). Results show the studied Kazakhstan *P. multocida* strains belong to capsular type B, and they formed a single clonal grouping with isolates ST64 (Pm240), ST44 (PM30), ST45 (PM36), ST46 (PM1192), ST44 (PM1200) and ST47 (DICM12/00359) extracted from diseased animals with haemorrhagic septicaemia in Hungary, Burma, Sri Lanka, Pakistan and Spain.

**Table 1:** Characterization of strains of *Pasteurella multocida* according to the MLST database

Strain/isolate Pasteurella/Saigas/	Country	Host	Disease	Capsule type	id	ST	No. of alleles							Reference
							*adk*	*aroA*	*pgi*	*g6pd*	*gdhA*	*mdh*	*deoD*	
2010/ZKO/KZ	Kazakhstan	Saiga	Unconfirmed	B	1362	64	26	28	25	23	6	22	23	In this study
2011/ZKO/KZ	Kazakhstan	Saiga	Unconfirmed	B	1363	64	26	28	25	23	6	22	23	In this study
2012/Kostanai/KZ	Kazakhstan	Saiga	Unconfirmed	B	1364	64	26	28	25	23	6	22	23	In this study
2015/Akmola/KZ	Kazakhstan	Saiga	HS	B	1365	64	26	28	25	23	6	22	23	In this study
2015/Kostanai/KZ	Kazakhstan	Saiga	HS	B	1366	64	26	28	25	23	6	22	23	In this study
Pm240	Hungary	bovine	HS	B	193	64	26	28	25	23	6	22	23	[21]
PM30	Burma	bovine	HS	B	122	44	26	27	23	23	22	22	25	[25]
PM36	Africa	bovine	HS	E	123	45	26	28	24	23	6	22	26	[25]
PM1192	Sri Lanka	bovine	HS	B	124	46	26	27	25	23	22	22	25	[25]
PM1200	Pakistan	bovine	HS	B	125	44	26	27	23	23	22	22	25	[26]
DICM12/00359	Spain	porcine	PMS	B	126	47	26	28	25	23	23	22	23	[27]
P55	Hungary	porcine	PMS	B	170	61	26	29	23	6	23	22	25	[50]

Of them the closest by 7 alleles (adk, aroA, deoD, pgi, g6pd, mdh, gdhA) to strains of *P. multocida*, isolated from the saigas in Kazakhstan was ST64 (id: 193) isolated from the bovine with septicaemia in Hungary.

### Virulence-associated gene profile

The molecular genetics technique multiplex PCR for detection of virulence-associated genes was applied to the isolates from saigas.

All studied isolates contained genes encoding outer membrane proteins (*ompA, ompH, oma87, plpB*), adhesins (*hsf1, hsf2*), iron uptake genes (*fimA, ptfA, exbB, tadD, fur*) and enzymes (*nanH, nanB, pmHAS*). Gene *toxA*, encoding dermonecrotic toxin was not detected in the studied *P. multocida* isolates.

## Discussion

It appears saiga antelopes are carriers of the *Pasteurellaceae* family in common with a wide range of mammals and birds, domestic and wild. In different years bacteria of the family Pasteurellaceae were isolated both from healthy and diseased antelope. Saiga mass mortalities were reported in 2010 in West Kazakhstan region and in 2015 in Kostanay region of Kazakhstan. Mass mortality events, where bacteria of the family Pasteurellaceae were detected, occurred in 1981, 1984, 1988 and 2015. Similarly, during smaller die-offs bacteria of this family were also isolated from samples. This includes 500 saiga which died on the same site and during the same season as the deaths in the West in 2010 but in the following year and, a thousand saiga which died in Kostanay region in 2012 after calving. Five *P. multocida* strains were isolated from dead saigas from each of these epidemic sites and they are described and characterized in the present study.

Definitive diagnosis of HS was only possible in 2015 and the aetiology ascribed to *P.multocida* serotype B, the isolated pathogen. This diagnosis of HS was based on clinical signs, post mortem examination, histology and microbiology with no other pathogen isolated or detected consistently in all cases examined other than *P. multocida* including by next generation sequencing (unpublished). The literature has only incomplete phenotypic data of Pasteurella isolated from saigas [14, 15] and genotypic data of *Pasteurella* isolated from saigas are not available and this is a priority for future research.

Morphological and biochemical characterization showed that the isolates belonged to *P. multocida* genus of the *Pasteurellaceae* family. Genetic studies have confirmed the findings of phenotypic characterization. PCR for KMTI gene fragment resulted in amplification of PCR product characteristic for *P. multocida*.

Differences between *P. multocida* subspecies multocida, septica and gallicida can be seen through the use of sorbitol, trehalose, and fermentation reactions but are usually not clear. Currently, ribotyping based on sequence analysis of ribosomal RNA 16S gene is a very effective tool for the differentiation of strains of the family *Pasteurellaceae* [22].

Phylogenetic analysis of ribosomal RNA 16S gene showed that all five isolates were identical to the strains of *P. multocida Subsp. multocida* associated with haemorrhagic septicemia in cattle, sheep and pigs with 100% homology. These data confirmed the data obtained by Dey et al., who conducted comparative analysis of 16S rRNA gene sequences and showed 99.9% nucleotide sequence identity amongst *P. multocida* serogroup B strains isolated from hemorrhagic septicemia diseased buffalo, cattle, pigs, sheep and goats [23]. The observation that over 27 years this line of bacteria on the studied site of a genome in the species saiga has not changed is of considerable interest and this should be tracked in future isolates.

There is little information about the type of capsules of *P. multocida*, isolated from wild animals. Strains of *P. multocida*, isolated from wild animals differ depending on the location and the host. Strains of capsular type A are often associated with respiratory tract infections and type B strains mainly cause septicemia in wild animals [24, 25]. PCR capsular typing of *P. multocida* strains isolated from saigas showed that all isolates belonged to type B. Analysis of ribosomal RNA 16S gene, and capsular genotyping gave reason to assume that these *P. multocida* isolates were similar to pathogens that are associated with haemorrhagic septicemia in buffalo, cattle, sheep and pigs [26–28].

MLST analysis showed that the studied strains of *P. multocida* from saigas are of the capsule type B and form one clonal group with isolates ST64 (Pm240), ST44 (PM30), ST45 (PM36), ST46 (PM1192), ST44 (PM1200). The studied strains of P. multtocida from saigas were 100% identical with the strain ST64 (Pm240) isolated from bovine with hemorrhagic septicemia in Hungary.

However, these data are insufficient to understand, completely, the epidemiology of pasteurellosis infection and study of the virulence-associated whole genetic profiles of *P. multocida* bacteria are needed.

*P. multocida* pathogenicity for various animal species has been shown to be associated with various so-called virulence factors [29] and various researchers suggested different genes that may be responsible for the morbidity of different species and therefore these can be used as epidemiological markers [30–32].

Gene toxA, encoding dermonecrotic toxin was not detected in the studied *P. multocida* strains isolated from saiga. There is evidence that high prevalence of the gene toxA was observed mainly in type A and D [30–33]. Harper, M. et al. [34] in their studies showed that the gene toxA, encoding dermonecrotic toxin is mainly characteristic for serogroup D and plays an essential role in the pathogenesis of atrophic rhinitis of pigs. Other authors have shown that the gene toxA is characteristic for serogroup A and is responsible for respiratory disease of sheep and goats [30, 31, 34].

All studied isolates from saigas contained a combination of genes associated with respiratory diseases of other animals (ptfA, ompA, ompH, oma87, plpB, fimA, hsf-2, pfhA, exbB, tonB, hgbA, fur, nanB, nanH and pmHAS). Studies conducted by Ewers et al. [31], showed that pfhA gene plays a great role in the pathogenesis of respiratory diseases of cattle, and in combination with toxA gene it causes disease in pigs [31]. Other authors showed that disease in cattle was connected directly with ptfA gene [32]. Therefore, pfhA and ptfA genes occupy a special place in the pathogenesis of *P. multocida*. Given the important characteristics of pfhA and ptfA genes, the authors recommend them as a marker for the epidemiological characterization of field strains and isolates of *P. multocida*.

## Conclusion

This is the first complete description of the phenotypic and genetic characteristics of *P. multocida* strains isolated from saigas associated with opportunistic isolation, sporadic cases of disease and peracute mass mortality events. The isolated agents were typed using classical and modern methods and virulence-associated genes identified, which could be used as epidemiological markers in future studies but whole genome sequences may present better opportunities. These data suggest that the same bacteria are present in the saiga at the time of death whether as an opportunistic pathogen and cause of death or as an incidental finding and/or post mortem invader. These bacteria are present in animals at the population level and there seems very little variation phylogenetically over space and time. Activation of virulence and passing the mucosa into the blood stream is required prior to septicaemia and the capsular typing confirmed the organisms to be of a serotype B often associated with HS and this supported the diagnosis in 2015. Without sufficient pathological and epidemiological supporting evidence, the reported pasteurellosis described in this study remain of uncertain aetiology except for the HS in 2015. *P. multocida* can be a factor in other respiratory disease syndromes of mild to severe nature sometimes associated with co-infections and this might be the explanation of the different epidemiologies and pathologies observed. The fact that all isolates were similar yet the pathologies different suggests that the pathogen is not acting alone and the conditions precipitating a variety of syndromes affecting saiga antelope may differ. Isolation, phenotypic and genetic characterization of the isolates can be used in the understanding of the epidemiology of pasteurellosis infection in saigas and predicting virulent potential of these opportunistic bacteria. In order to more fully understand the observed syndromes, the microbiome of saigas should be studied and immunity to the pathogen and or its commensal relationship. Definitive diagnosis is supported by integrating studies at the molecular level with epidemiological and pathological investigations in both healthy and sick saigas.

## Methods

### Field sample collection and transport

**Fig. 2:**
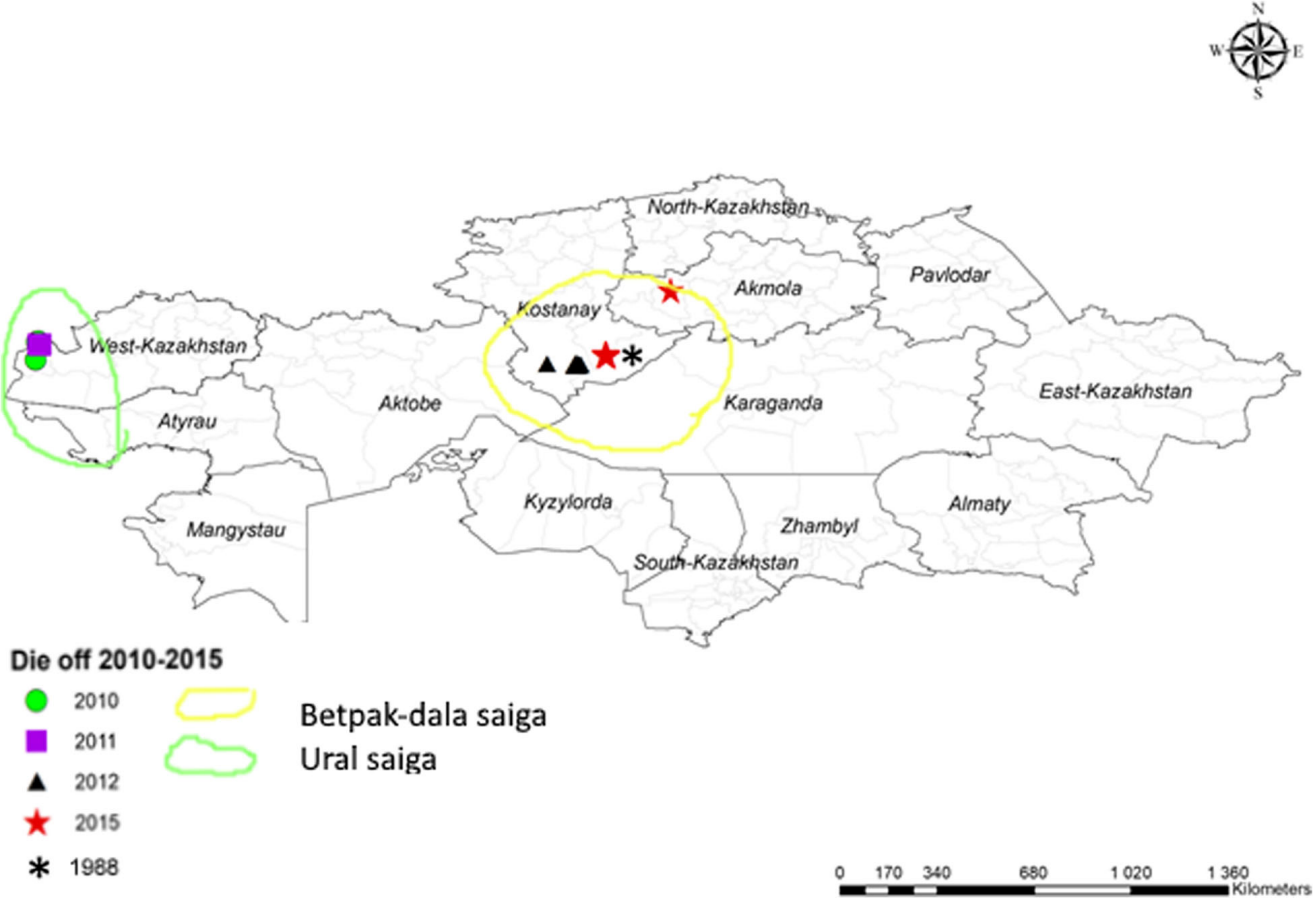
Map of Kazakhstan. Explanation: Map of Kazakhstan and its provinces (Oblasts) showing the approximate location of the carcasses sampled between 2010 and 2015. Including two geographically separate populations of saiga, the Ural and Betpak-dala. (Note that the die off in 2010 and 2011 were in the exact same geographical position but to show both the locations graphically, they are slightly separated on the map). To reproduce the physical/geographic part of the map permission is not required

Biological samples from dead saigas were collected in the West Kazakhstan, Kostanay and Akmola oblasts as soon after death as possible in 2010–2015 with a range of minutes to several days post mortem. Samples taken from each animal were placed separately in cryovials and delivered to the RIBSP frozen in liquid nitrogen. Detailed information on the place of death of saigas, the number of dead animals, clinical signs, pathoanatomical changes and samples taken are given in Table 2. Locations of sampling of saigas are shown in Fig. 2. On arrival at the laboratory the samples were maintained at -20 °C until defrosted for analysis.

**Table 2:** Details of sampling for bacteriology from mass mortality events in saiga 2010–2015

Location	Date	Population/Mortality (% of those who died by sex and age)	Clinical signs	Pathology	Number of animals/samples
Borsy, West Kazakhstan	19–29 < May, 2010	25,000 / ~ 12,000 (Female - 64,0%, male – 0,4%, calves – 35,6%)	According to witnesses and video material: Depression, respiratory distress, bloody froth from nose, saliva discharge mouth, occasional bloat and diarrhoea, rapid death.	Carcasses were 1–2 days old. Pathology incomplete – probe samples.	3/blood, spleen, lymph nodes, lungs
Borsy, West Kazakhstan	20–29 < May, 2011	10,000 / ~ 500 (Female - 98,6%, male – 1,4%)	According to witnesses and video material: similar to above	Carcases were 1–2 days old. Pathology incomplete – probe samples.	2/blood, spleen, lymph nodes, lungs
Zhangeldinsky region, Kostanay	17–30 < May, 2012	40,000 / ~ 1000 (Female - 93,3%, male – 6,7%)	Depression, discharge from the nose and mouth, diarrhoea	haemorrhages subcutaneous tissue, lungs, endocardium but pathology incomplete –probe samples from fresh carcases.	14/blood, spleen, lymph nodes, lungs, liver, kidneys
Zholoba, Kostanay	< 11–19 May, 2015	70,000 / ~ 70,000 (Female - 98,2%, male - 1,8%, calves all died)	Depression, weakness, ataxia, frothy nasal fluid and/or saliva from the mouth, terminal diarrhoea, rapid death.	haemorrhages sub-cutaneous - serosal surfaces, epi- and endocardium, lung congestion and oedema, enteric congestion and catarrh, liver and kidney congestion, enlarged gall bladder	26/blood, spleen, lymph nodes, lungs
Ortakara, Kostanay,	< 20–26 May, 2015	8000 / ~ 8000 (Female - 90,2%, male −9,8%, calves all died)	Depression, weakness, ataxia, occasional nasal/salivary fluid, terminal bloody diarrhoea, rapid death.	haemorrhages sub-cutaneous- serosal surfaces, epi-and endocardium, lung congestion and occasional oedema, haemorrhagic lymph nodes, enteritic congestion and catarrh, liver and kidney congestion, enlarged gall bladder	6/blood, spleen, lymph nodes, lungs

### Detection and identification of pathogens

To find out the causes of the disease and the death of saigas in 2010–2015, we conducted laboratory studies of collected samples of biological material. All delivered samples were examined for viruses, bacteria and blood-parasites.

The detection of viruses, bacteria and parasites in PCR was carried out using the primers described in the literature for pasteurella [35], mycoplasma spp. [36], Clostridium toxins [37], Coxiella [38], Visna Maedi viruses [39], Akabane viruses [40], MCF viruses [41], Theileria [42], Babezii [43], Anaplasma [44].

Detection of viruses of peste des petits ruminants, foot-and-mouth disease, bluetongue, sheep pox has been carried out using commercial kits, according to the manufacturer’s instructions (Tetracore, USA). Amplification and analysis of its products were performed in the LightCycler 2.0 (Roche). Isolation and identification of *Listeria monocytogenes* was performed based on PCR method using commercial kit (Lister, AmpliSens®). To detect anthrax DNA the kit Bacillus Anthax Real Time PCR Kit (Liferiver, China) was used.

### Isolation and identification of Pasteurella isolates

Smears from inner organs and from broth and agar cultures were stained by Romanovsky-Giemsa, Gram and Loeffler methylene blue [19].

Isolation of *Pasteurella* was implemented by cultivation of pathological material samples in meat-broth (MPB), Hottinger digest broth and plain agar (IPA) with 5% sterile horse serum.

Stained smears confirmed the organism a rod, coccobacilli. Sub-culturing was undertaken from blood and MacConkey agar primary plates. Bacterial colonies with the following characteristics; rough or smooth, and which were Gram negative, with or without growth on MacConkey, presence or absence haemolysis on blood agar, were selected. Then each bacterial colony was characterised and further bacterial identification completed using a series of primary and secondary biochemical tests following standard procedures [20].

DNA extraction was performed on isolates of *P. multocida* after PCR results using a commercial kit “PrepMan Ultra Sample Preparation Reagent” by Applied Biosystems (USA), according to the manufacturer’s protocol.

Identification of the isolated Pasteurella culture was conducted according to biochemical indicators such as saccharolytic and proteolytic enzymes excretion, formation of catalase, acetyl-methyl-carbinol, ammonia, hydrogen sulfide, indole, lysis of red blood cells and cattle bile, nitrate reduction [19].

### Ribotyping

Pasteurella strains ribotyping was performed using the following primers P16Sf: 5/-AGAGTTTGATYMTGGC-3/ and P16Sr: 5/-GYTACCTTGTTACGACTT-3/ [22]. PCR mixture consisted of 1 x PCR buffer, and 4 mM MgCl_2_ 1,25 U Taq DNA polymerase, 200 mM dNTP, 10 pmol of each primer and 2.5 μl of the DNA sample under study. Initial denaturation was held at 95 °C for 5 min followed by 35 cycles of denaturation at 94 °C for 30 s, annealing at 55 °C for 30 s, and replication at 68 °C for 1 min. Post-PCR replication was held at 68 °C for 7 min. Detection of PCR products was conducted in 2% agarose gel in 1 x TAE buffer containing ethidium bromide.

### *Pasteurella multocida* capsular typing

Typing of the strains was conducted with the use of PCR analysis applying capsular-specific primers for determining capsular groups A, B, D, E, F [45].

### Multi locus sequence typing (MLST)

For the MLST analysis a set of seven “housekeeping” genes were chosen for *P. multocida* bacteria based on their localization and across various functions of the chromosome, for comparison and determination of strains with unique allelic profiles from the database pubmlst.org and results are shown in Table 3.

**Table 3:** Characteristics of the primers for the 7 loci of bacterium *P. multocida*

№	Loci	Loci Function	Primer sequences		Product size (bp)
			Forward	Reverse	
1	*аdk -*adenylate cyclase	nucleotide biosynthesis	AAGGBACWCAAGCVCAAT	CACTTTTTKYGTMCCGTC	531
2	*aroA -* 3-phosphoshikimate 1-carboxyvinyl transferase	amino acid biosynthesis	TTTACCDGGYTCYAAAAG	CTTTYACVCGCCAGTTAT	558
3	*pgi -* phosphoglucose isomerase	energy metabolism; glycolysis	GCCWGTGYTKGTTGATGG	TTGKGCTGGCGCRATRAA	609
4	*g6pd -* glucose-6-phosphate 1-dehydrogenase	energy metabolism: pentose	CHGGYGAYYTMACTYATCG	TTTBGCGATBARTTTRTCRGC	513
5	*gdhA -* glutamate dehydrogenase	amino acid biosynthesis	YTTAGTTGARCCTGAACG	CTTGACCTTCAATYGTGC	651
6	*mdh -* malate dehydrogenase	energy metabolism; TCA cycle	AAGTTGCWGTWYTAGGTG	CCTAATTCAATATCYGCACG	552
7	*deoD -* purine nucleoside phosphorylase	nucleotide biosynthesis	GTGCATTTGCYGATGTTG	TGSYGTKGTTTGTTCGTG	576

Processing of the PCR products was performed using a set of AmpliTaq Gold (Applied Biosystems). The reaction mixture contained 25 μl 2x AmpliTaq Gold buffer, 1 μl of each 20 pmol primers, 2 μl of the DNA and distilled water up to 50 μl. The cycling conditions consisted of initial denaturation at 95 °C for 5 min followed by 35 cycles of 94^0^c for 45 s, 56-60 °C for 45 s, 72 °C for 2 min, and a final extension at 72 °C for 10 min [46].

MLST analysis was completed on strains of *P. multocida*, isolated from the saigas on the territory of the Republic of Kazakhstan in 2010–2015. Kazakhstan strains of *P. multocida* were confirmed through PCR products and studied allelic profiles: adk, aroA, deoD, pgi, g6pd, mdh and gdhA, according to the database pubmlst.org.

### Identification of virulence genes

For amplification of *P. multocida* virulence genes using PCR we used AccuPrime Taq DNA Polymerase High Fidelity kit (Invitrogen) and conducted multiplex PCR for rapid and simultaneous detection of virulence genes of *P. multocida* [29]. To identify fragments of virulence factors in PCR, primers were used for toxA, pfhA, ompH, oma87, nanB, nanH, pmHAS, ompA, plpB, fimA, hsf-2, exbB, tonB, fur, ptfA, hgbA [47].

### Sequencing

Sequencing of the PCR products was implemented using the Big Dye 3.1 terminator sequencing kit on an automated 16-capillary sequencer Genetic Analyser 3130 xl, Applied Biosystems, USA.

### Phylogenetic analysis

Phylogenetic analysis was conducted in MEGA6 [48]. A phylogenetic tree was generated using the Maximum Parsimony method. The tree was obtained using the Subtree-Pruning-Regrafting (SPR) algorithm [49] with search the initial level in which the initial trees were obtained by the random addition of sequences. The tree is drawn to scale, with branch lengths calculated using the average pathway method and are in the units of the number of changes over the whole sequence. There were a total of 1480 positions in the final dataset.

The nucleotide sequence of ribosomal RNA 16S gene was determined by direct sequencing and the comparative analysis was implemented with the various species and subspecies of Pasteurella genus bacteria available in Genbank. For the comparative analysis, the nucleotide sequences of the 16S rRNA gene of the following Pasteurella bacterium were used: *P. multocida* Subs*p. multocida* strain PM82 (DQ288145), *P. multocida* Subs*p. multocida* strain P52 (DQ286927), *P. multocida* Subs*p. multocida* strain PM104 (AY299311), *P. multocida Subsp. multocida_XJNKY-124YF1 (JX984984), P. multocida Subsp. multocida_Clin36 (EF579814), P. multocida* Subs*p. multocida*_E348/08 (HM746978), *P. multocida* Subs*p. multocida*_CCUG 17976 (NR041809), *P. multocida* Subs*p. multocida* (AF294410), *P. multocida* Subs*p. multocida* NCTC 10322 (AY078999), *P. multocida* Subs*p. multocida* NCTC 10322 (NR115137), *P. multocida* subsp. *septica* strain D514 (AF294422), *P. multocida* subsp. *gallicida*_strain 77,179 (AF326324), *P. multocida* subsp. *septica*_PM24 (NR115138), *P. multocida* subsp. *septica*_strain D496 (AF294421), *P. multocida* subsp. *septica*_strain D755 (AF294423).

## Abbreviations

DNA: Deoxyribonucleic acid
FMD: Foot and mouth disease
HS: Hemorrhagic septicemia
MLST: Multi Locus Sequence Typing
NCBI: National Center for Biotechnology Information
PCR: Polymerase chain reaction
PPR: Peste des petits ruminants
RNA: Ribonucleic acid
